# Cyanobacterial Oxygenic Photosynthesis is Protected by Flavodiiron Proteins

**DOI:** 10.3390/life5010716

**Published:** 2015-03-09

**Authors:** Yagut Allahverdiyeva, Janne Isojärvi, Pengpeng Zhang, Eva-Mari Aro

**Affiliations:** Molecular Plant Biology, Department of Biochemistry, University of Turku, FI-20014 Turku, Finland; E-Mails: allahve@utu.fi (Y.A.); jaheis@utu.fi (J.I.); zhangpengpeng@caas.cn (P.Z.)

**Keywords:** flavodiiron protein, flavoprotein, cyanobacteria, Mehler-like reaction, nitrogenase, photosystem, photodamage, electron transfer, photosynthesis, phycobilisome, photoprotection

## Abstract

Flavodiiron proteins (FDPs, also called flavoproteins, Flvs) are modular enzymes widely present in Bacteria and Archaea. The evolution of cyanobacteria and oxygenic photosynthesis occurred in concert with the modulation of typical bacterial FDPs. Present cyanobacterial FDPs are composed of three domains, the β-lactamase-like, flavodoxin-like and flavin-reductase like domains. Cyanobacterial FDPs function as hetero- and homodimers and are involved in the regulation of photosynthetic electron transport. Whilst Flv2 and Flv4 proteins are limited to specific cyanobacterial species (β-cyanobacteria) and function in photoprotection of Photosystem II, Flv1 and Flv3 proteins, functioning in the “Mehler-like” reaction and safeguarding Photosystem I under fluctuating light conditions, occur in nearly all cyanobacteria and additionally in green algae, mosses and lycophytes. Filamentous cyanobacteria have additional FDPs in heterocyst cells, ensuring a microaerobic environment for the function of the nitrogenase enzyme under the light. Here, the evolution, occurrence and functional mechanisms of various FDPs in oxygenic photosynthetic organisms are discussed.

## 1. Introduction

Flavodiiron proteins (FDPs), previously called A-type flavoproteins (Flv) [1], are a large family of enzymes sharing sequence similarity. FDPs have been found mainly in anaerobic and some aerobic prokaryotes (Bacteria including cyanobacteria, and Archaea), and in Protozoa. Data mining of sequenced genomes has also led to the discovery of FDP homologs in some photosynthetic eukaryotes [2,3].

All FDPs share two conserved structural domains: the N-terminal metallo-*β*-lactamase-like domain, harboring a non-heme diiron center where O_2_ and/or NO reduction take place; and the C-terminal flavodoxin-like domain, containing a flavin mononucleotide (FMN) moiety [4,5]. X-ray crystallography of FDPs from different organisms has provided valuable data for the elucidation of the electron transfer properties in the active site during O_2_ and/or NO reduction [6,7,8,9]. The functional form of FDPs in anaerobic prokaryotes and eukaryotic protozoa has been resolved as a homodimer or homotetramer arranged in a “head to tail” configuration so that the diiron center of one monomer and the FMN in the other monomer closely contact each other, which ensures fast electron transfer between the cofactors.

**Figure 1:**
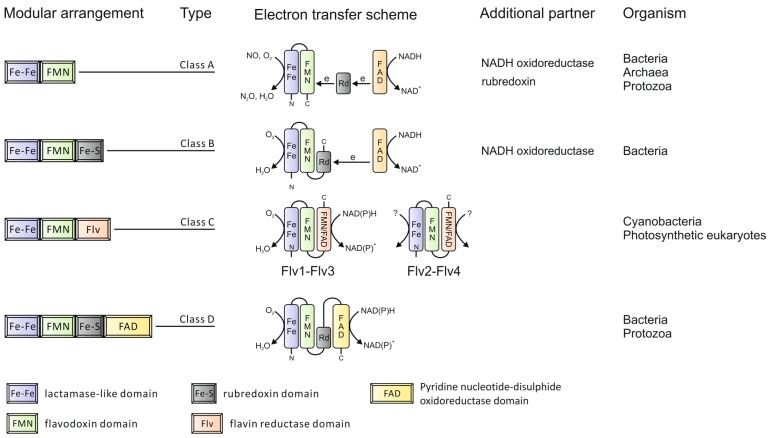
Modular organization of the Flavodiiron protein (FDP) family.

In addition to the common sequence core, some FDPs also have C-terminal extensions. Based on these C-terminal extensions, FDPs can be grouped into four classes [5] as depicted in Figure 1. The majority of FDPs belong to Class A, which is the simplest type with the shortest extension sequences, representing the minimal core structure. These can be found in Bacteria, Archaea and Protozoa. Class B FDPs are found in Enterobacteria, whilst Class D FDPs are present in some Bacteria and Protozoa. Class C FDPs seem to be specific to oxygenic photosynthetic organisms. The additional flavin reductase-like domain in this specific class makes it possible for nicotinamide adenine dinucleotide (phosphate), reduced form (NAD(P)H) to be directly used as an electron donor. The extension component is also coupled with extra cofactor(s), which bring additional features to FDPs during electron transfer. The number of redox partners required during electron transfer depends on the modular arrangement of FDPs. The more complex the FDPs, the fewer partners are involved. For example, rubredoxin donates electrons to many FDPs from Class A but is not needed for FDPs from Class B, which have a rubredoxin domain fused in the polypeptide.

The gene organization of FDPs in different organisms indicates a complex evolution. For example, a rubredoxin electron donor of *Desulfovibrio gigas* rubredoxin:oxygen oxidoreductase, Dg_ROO (Class A FDP), is encoded in the same operon as ROO [10] and the *Escherichia coli* flavorubredoxin (Class B FDP) and its partner nicotinamide adenine dinucleotide, reduced form (NADH): flavorubredoxin oxidoreductase are encoded by the same operon. Similar gene organizations have also been found in other Bacteria and Archaea. More interestingly, proteins homologous to the flavin-reductase domain of Class C FDPs are detected in some Bacteria and Archaea, but they are not in the same cistronic unit as FDPs. An example of the latter can be found in *Nodularia spumigena* CCY 9414, whose flavin-reductase-like protein is encoded directly downstream of a Class A FDP gene within the same operon. Thus, the complexity of the FDPs may result from multiple genome rearrangements and gene fusions during evolution. 

The FDPs from anaerobic species have been proposed to protect against both O_2_ and/or NO toxicity by catalyzing the final step of O_2_ and/or NO reduction. It is worth mentioning that some FDPs act preferably as NO-reductases [11,12], others as O_2_-reductase [6,8,13], whereas some FDPs can catalyze both reactions [14]. Additionally, there seems to be functional relationship between respiratory terminal oxidases and the catalytic activity of FDPs. In *Giardia*, which lacks both the respiratory oxidases and reactive oxygen species (ROS) scavenging enzymes, FDP shows a high O_2_-reductase activity (>40 s^−1^), but very low NO-reductase activity (~0.2 s^−1^) [6]. In organisms containing respiratory oxidases (*Escherichia coli*, *Desulfovibrio gigas*, *Desulfovibrio vulgaris*, *Moorella*), FDPs are known to have either strict NO-reductase function or dual function, thus cooperating with respiratory oxidases in protection against O_2_ toxicity.

## 2. *flv* Genes in Oxygenic Photosynthetic Organisms

### 2.1. The flv Gene Family and its Organization in Cyanobacterial Genomes

**Figure 2:**
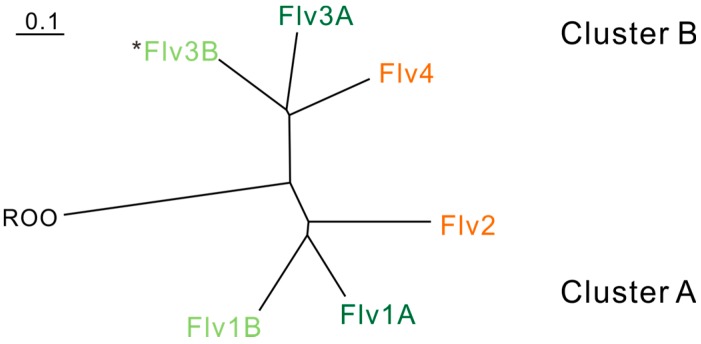
Relationship between cyanobacterial FDPs based on sequence similarity and physiological functions. Phylogenic analysis is based on FDPs in *Anabaena* sp. PCC 7120. ROO (rubredoxin:oxygen oxidoreductase) from *Desulfovibrio gigas* is used as an outgroup. The FDPs functioning in the photoprotection of Photosystem II are indicated in orange. The FDPs functioning in the Mehler-like reaction and protection of Photosystem I are indicated in green. The FDPs indicated in dark green function in vegetative cells, and those in light green function in heterocysts. *Flv3B can be arranged and function as a homodimer [18].

Genes encoding FDPs (*flv*) can be found in most sequenced cyanobacteria, including the obligatory photoautotrophic species. Different cyanobacterial strains may possess several copies (2–6) of *flv* genes, thus comprising a small family encoding different FDPs. We have earlier shown that FDPs in oxygenic photosynthetic organisms can be grouped into almost symmetrical clusters (cluster A, including Flv1 and Flv2, and cluster B, including Flv3 and Flv4) and appear in pair(s) (*flv1*-*flv3* or *flv2*-*flv4*) [2]. The distribution of the clusters is depicted in Figure 2 and the number of *flv* genes and their organization in the genomes of oxygenic photosynthetic organisms are summarized in Table 1. Cyanobacteria can be divided into two groups depending on RubBisCO and carboxysome types: α- and β-cyanobacteria [15,16,17]. The α-cyanobacteria have only one pair of *flv* genes (*flv1*-*flv3*). Some unicellular and filamentous, non-heterocystous β-cyanobacteria, including the model unicellular cyanobacterium *Synechocystis* sp. PCC 6803, possess the *flv2*-*flv4* pair in addition to *flv1*-*flv3*. All heterocystous filamentous β-cyanobacteria contain two pairs of *flv1*-*flv3*, designated as *flv1a*-*flv3a* and *flv1b*-*flv3b*, thus having 4 or 6 *flv* genes depending on the presence of the *flv2*-*flv4* pair. It seems that the *flv1*-*flv3* pair is largely present in all organisms containing C Class FDPs, whereas the *flv2*-*flv4* pair is present only in some β-cyanobacteria.

A further survey of the *flv* gene loci indicated that the genes encoding many of the *flv* paralog pairs are localized sequentially in genomes. Usually, the *flv2* and *flv4* genes are organized as an operon consisting of three genes arranged as in tandem orientation in the same direction of transcription (*flv4-ORF-flv2*). The structure of the *flv4-ORF-flv2* operon is highly conserved, except for *Microcoleus* sp. PCC 7113, which possesses five ORFs between *flv2* and *flv4* genes (Table 1). In some cases, *flv4* and *flv2* are also located far away from each other and probably do not organize an operon.

The organization of the *flv1* and *flv3* genes is not conserved. The majority of α-cyanobacteria arrange *flv1* and *flv3* in the *flv3-flv1* operons, whilst in β-cyanobacteria, the genes might be organized as *flv3a-flv1a* operons*,* or separated by 1–5 ORF(s) (designated as *flv3a-ORF(s)-flv1a* in Table 1), or otherwise be spread out in the genome. In contrast, the *flv1b* and *flv3b* genes are always arranged together in the *flv3b*-*flv1b* operon and are likely co-transcribed, as demonstrated in *Anabaena* sp. PCC 7120 [19]. In the genomes of eukaryotes, *flv3-flv1* are not usually clustered together. However, some eukaryotes retain the clustering. For instance, *Paulinella chromatophora*, the photosynthetic protozoa bearing photosynthetic entities (chromatophores), derived from cyanobacteria [20]. In some green algae, *flv1*-*flv3* also retain clustering, even though ORF(s) exist in between.

**Table 1:** Genes encoding FDPs in cyanobacteria and photosynthetic eukaryotes.

	No. of *flv*'s	*flv1(a)*	*flv3(a)*	*flv2*	*flv4*	*flv1b*	*flv3b*	Gene organization (*flv1(a), flv3(a*))	Gene organization (*flv2, flv4*)	Gene organization (*flv1b, flv3b*)
**α-Cyanobacteria (unicellular)**										
*Cyanobium gracile* PCC 6307	2							*→flv3-flv1→*		
*Prochlorococcus marinus* AS9601	2							*→flv3-flv1→*		
*Prochlorococcus marinus* MED4	2							*→flv3-flv1→*		
*Prochlorococcus marinus* MIT 9202	2							*→flv3-flv1→*		
*Prochlorococcus marinus* MIT 9211	2							*→flv3-flv1→*		
*Prochlorococcus marinus* MIT 9215	2							*→flv3-flv1→*		
*Prochlorococcus marinus* MIT 9301	2							*→flv3-flv1→*		
*Prochlorococcus marinus* MIT 9303	2							*		
*Prochlorococcus marinus* MIT 9312	2							*→flv3-flv1→*		
*Prochlorococcus marinus* MIT9313	2							*→flv3-flv1→*		
*Prochlorococcus marinus* NATL1A	2							*→flv3-flv1→*		
*Prochlorococcus marinus* NATL2A	2							*→flv3-flv1→*		
*Prochlorococcus marinus* SS120	2							*→flv3-flv1→*		
*Synechococcus* BL107	2							*→flv3-flv1→*		
*Synechococcus* CB0101	2							*		
*Synechococcus* CB0205	2							*→flv3-6 ORF's flv1→*		
*Synechococcus* CC9311	2							*→flv3-flv1→*		
*Synechococcus* CC9605	2							*→flv3-WP_011365453-flv1→*		
*Synechococcus* CC9902	2							*→flv3-2 ORF's-flv1→*		
*Synechococcus* RCC307	2							*→flv3-flv1→*		
*Synechococcus* RS9916	2							*→flv3-WP_007099263-flv1→*		
*Synechococcus* RS9917	2							*→flv3-flv1→*		
*Synechococcus* WH 7805	2							*→flv3-flv1→*		
*Synechococcus* WH 8102	2							*→flv3-flv1→*		
*Synechococcus* WH 8109	2							*→flv3-flv1→*		
*Synechococcus WH 5701*	2							*→flv-WP_00617255-flv1→*		
*Synechococcus WH 7803*	2							*→flv3-flv1→*		
**β-Cyanobacteria (unicellular)**										
*Acaryochloris marina* MBIC11017	2							*→flv3-AM1_1385-flv1→*		
*Chroococcidiopsis thermalis* PCC 7203	2							*→flv3-2 ORF's-flv1→*		
*Cyanobacterium aponinum* PCC 10605	2							*		
*Cyanobacterium stanieri* PCC 7202	2							*		
*Cyanobacterium* UCYN-A	0							*		
*Cyanothece* CCY 0110	4							*	*→flv4-WP_008278275-flv2→*	
*Cyanothece* PCC 7424	4							*	*→flv4-PCC7424_0480-flv2→*	
*Cyanothece* PCC 7425	2							*→flv3-WP_012627311-flv1→*		
*Cyanothece* PCC 7822	4							*	*→flv4-Cyan7822_3509-flv2→*	
*Cyanothece* PCC 8801	4							*	*→flv4-PCC8801_3605-flv2→*	
*Cyanothece* PCC 8802	4							*	*→flv4-Cyan8802_2509-flv2→*	
*Crocosphaera watsonii* WH 8501	2							*		
*Dactylococcopsis salina* PCC 8305	2							*→flv3-flv1→*		
*Gloeobacter kilaueensis* JS1	2							*→flv3-flv1→*		
*Gloeobacter violaceus* PCC 7421	2							*→flv3-flv1→*		
*Gloeocapsa* PCC 7428	2							*→flv3-flv1→*		
*Halothece* PCC 7418	4							*	*→flv4-PCC7418_1461-flv2→*	
*Microcystis aeruginosa* NIES-843	4							*	*→flv4-YP_001660097-flv2→*	
*Microcystis aeruginosa* PCC 7806	4							*	*→flv4-IPF_2587-flv2→*	
*Pleurocapsa* PCC 7327	4							*→flv3-Ple7327_0831-flv1→*	*→flv4-Ple7327_3773-flv2→*	
*Stanieria cyanosphaera* PCC 7437	4							*	*→flv4-Sta7437_3860-flv2→*	
*Synechococcus* PCC 7335	4							*	*	
*Synechococcus PCC 7002*	2							*		
*Synechococcus PCC 6312*	2							*		
*Synechococcus PCC 7502*	2							*		
*Synechococcus JA-3-3Ab*	2							*		
*Synechococcus* JA-2-3B'a(2-13)	2							*		
*Synechococcus elongatus* PCC 7942	2							*→flv3-flv1→*		
*Synechococcus elongatus* PCC 6301	2							*→flv3-flv1→*		
*Synechocystis* PCC 6803	4							*	*→flv4-sll0218-flv2→*	
*Synechocystis* PCC 6714	2							*→flv3-2 ORF's-flv1→*		
*Thermosynechococcus* NK55a	2							*		
*Thermosynechococcus elongatus* BP-1	2								*	
**β-Cyanobacteria (filamentous)**										
***Anabaena* 90**	4							*→flv3-WP_015078091-flv1→*		*→flv3b-flv1b→*
***Anabaena* PCC 7120**	6							*→flv3a-all3892,all3893,all3894-flv1a→*	*→flv4-all4445-flv2→*	*→flv3b-flv1b→*
***Anabaena cylindrica* PCC 7122**	4							*→flv3-flv1→*		*→flv3b-flv1b→*
***Anabaena variabilis* ATCC 29413**	6							*→flv3a-flv1a→*	*→flv4-Ava_1370-flv2→*	*→flv3b-flv1b→*
*Arthrospira platensis* NIES-39	2							*→flv3-flv1→*		
***Calothrix* 336/3**	4							*→flv3-Cal336_3958-flv1→*		*→flv3b–flv1b→*
***Calothrix* PCC 6303**	4							*→flv3-flv1→*		*→flv3b-flv1b→*
***Calothrix* PCC 7507**	6							*→flv3a-flv1a→*	*→flv4-Cal7507_5629-flv2→*	*→flv3b-flv1b→*
*Chamaesiphon minutus* PCC 6605	2							*→flv3-2 ORF's-flv1→*		
*Chloroflexus aurantiacus* J-10-fl	0									
***Cylindrospermopsis raciborskii* CS-505**	4							*→flv3-flv1→*		*→flv3b-flv1b→*
***Cylindrospermum stagnale* PCC 7417**	4							*→flv3-flv1→*		*→flv3b-flv1b→*
*Crinalium epipsammum* PCC 9333	2							*→flv3-YP_007142380-flv1→*		
*Geitlerinema PCC 7407*	2							*→flv3-flv1→*		
*Geminocystis herdmanii PCC 6308*	0									
*Leptolyngbya PCC 7376*	4							*	*→flv4-Lepto7376_3457-flv2→*	
*Lyngbya majuscula* 3L	2							*→flv3-flv1→*		
*Lyngbya* PCC 8106	2							*→flv3-WP_009783639-flv1→*		
*Microcoleus chthonoplastes* PCC 7420	4							*	*	
*Microcoleus* PCC 7113	4							*→flv3-3 ORF's-flv1→*	*→flv4-5 ORF's-flv2→*	
*Microcoleus vaginatus* FGP-2	2							*→flv3-EGK88546-flv1→*		
*Nodularia spumigena* CCY 9414 **	6							*	*→flv4-flv2→*	*→flv3b-flv1b→*
***Nostoc* PCC 7107**	6							*→flv3a-10 ORF's-flv1a→*	*→flv4-WP_015113616-flv2→*	*→flv3b-flv1b→*
***Nostoc* PCC 7524**	6							*→flv3a-6 ORF's-flv1a→*	*→flv4-Nos7524_2687-flv2→*	*→flv3b-flv1b→*
***Nostoc punctiforme* ATCC 29133**	5							*→flv3a-flv1a→*	*→Npun_R0592-flv2→*	*→flv3b-flv1b→*
***Nostoc azollae* 0708**	4							*→flv3-flv1→*		*→flv3b-flv1b→*
*Oscillatoria* PCC 6506	2							*		
*Oscillatoria acuminata* PCC 6304	2							*→flv3-flv1→*		
*Oscillatoria nigroviridis* PCC 7112	2							*→flv3-Osc7112_2977-flv1→*		
*Oscillatoriales* JSC-1	2							*		
*Planktothrix agardhii* NIVA-CYA 126/8	2							*→flv3-flv1→*		
*Pseudanabaena* sp. PCC 7367	4							*→flv3-flv1→*	*→flv4-Pse7367_3922-flv2→*	
*Raphidiopsis brookii D9*	2							*→flv3-flv1→*		
***Rivularia*** ** PCC 7116**	6							*→flv3a-flv1a→*	*→flv4-Riv7116_6032-flv2→*	*→flv3b-flv1b→*
*Trichodesmium erythraeum* IMS101	2							*		
**Photosynthetic protozoa**										
*Paulinella chromatophora*	2							*→flv3-flv1→*		
**Green algae**										
*Chlamydomonas reinhardtii*	2							*		
*Chlorella variabilis*	2							*		
*Micromonas pusilla* CCMP1545	2							*		
*Micromonas* RCC299	2							*		
*Ostreococcus lucimarinus* CCE9901	2							*→flv3-XP_001416099-flv1→*		
*Ostreococcus tauri*	2 (+2)^#^							*→flv3-3 ORF's-flv1→*		
*Volvox carteri f. nagariensis*	2							*		
**Land plants**										
*Dysgonomonas mossii* DSM 22836	1									
*Physcomitrella patens* subsp. patens	2							*		
*Selaginella moellendorffii*	2							*		
*Arabidopsis thaliana*	0									
Picea sitchensis***	1									
**Symbiodinium ^##^**										
*Symbiodinium Avir* (clade A1)	2							*		
*Symbiodinium FlAp1* (clade B1)	2							*		
*Symbiodinium Mf1.5b* (clade B1)	2							*		
*Symbiodinium Pd44b (* clade F1)	2							*		

The filamentous heterocystous cyanobacteria are marked with a gray background; * FDP-encoding gene orthologs are present but lacking gene organization; ** The FDP-encoding gene is splited into two genes, coding for a Class A FDP and flavin reductase, and situated sequentially in one polycistron; *** possess a Class A FDP; ^#^ possess two absolutely identical extra copies; ^##^ [21]

### 2.2. Expression and Regulation of flv Genes

A joint analysis of global transcriptomics and proteomics data can provide useful insights into the function of specific proteins and their regulation by environmental cues. *Synechocystis* sp. PCC 6803 has four genes (*sll1521*, *sll0219*, *sll0550* and *sll0217*) encoding FDPs (Flv1, Flv2, Flv3, and Flv4, respectively). Genome-wide DNA microarray data have shown that the transcription of the *flv2*, *flv4* and *flv3* genes of *Synechocystis* sp. PCC 6803 is strongly affected by various environmental factors (Table 2). Among all studied conditions, the most remarkable changes in *flv3*, *flv2* and *flv4* transcript levels were observed under Ci-limitation, Fe-depletion and different dark/light regimes. The transcript abundance of the *flv1* gene in *Synechocystis* sp. PCC 6803 is significantly lower under standard growth conditions [2]. Such a low abundance of *flv1* transcript and also of the Flv1 protein might result from the presence of antisense-RNA (as-RNA) for the *flv1* gene (as-*flv1*) [22]. However, more detailed expression studies of as-*flv1* under different environmental conditions would be required to make a strong conclusion. In contrast to other *flvs*, the *flv1* transcript level does not respond to high light or Ci-limitation (Table 2). Instead, a low induction in *flv1* transcript amount was observed under oxidative (methyl viologen and H_2_O_2_ treatment) and heat stress conditions (Table 2). Analysis of the *flv1* expression level by RT-PCR demonstrated 3.5 and 4.2 fold increases after 1 h and 12 h nitrosative stress treatments, respectively [23]. A differential expression pattern of *flv1* and *flv3* in *Synechocystis* sp. PCC 6803 is also consistent with the fact that, in this species, the *flv1* and *flv3* genes are spread out in the genome (Table 1)*.*

Differing from *Synechocystis* sp. PCC 6803, the *flv1a* and *flv3a* genes in *Anabaena* sp. PCC 7120 are clustered in the genome and this might suggest an operon organization (Table 1). The transcript level of *flv1a* is only slightly lower (3–4 times) than that of *flv3a* and the expression levels of both transcripts have been shown to respond positively to both high light and low CO_2_ treatment [24,25]. However, RNA-seq data [19,26] and transcript level analysis of the respective *flv* deletion mutants [27] suggest that *flv1a* and *flv3a* in *Anabaena* sp. PCC 7120 are likely transcribed independently. The extra pair of *flv* genes in *Anabaena* sp. PCC 7120, the *flv1b* and *flv3b* genes, which are arranged together in an operon and are likely co-transcribed [19], demonstrate clearly different expression profiles from the other *flv* genes in *Synechocystis* sp. PCC 6803 and *Anabaena* sp. PCC 7120. The transcript levels of *flv1b* and *flv3b* do not respond to changes in carbon or light regime, but substantially increase under N_2_-fixing conditions. The response is similar to that of the *nifH* gene, which encodes a subunit of the nitrogenase enzyme in heterocysts [24]. Interestingly, the *flv1b* transcript level remains somewhat lower than that of *flv3b*.

The significant up-regulation of Flv3 and Flv2 proteins under low CO_2_ levels, observed in Isobaric tags for relative and absolute quantitation (iTRAQ) shotgun analysis, is consistent with the above-mentioned microarray studies in *Synechocystis* sp. PCC 6803 (Table 1 and Table 3). Interestingly, the Flv3, and to some extent, also the Flv1 proteins, exhibited a low expression level under chemoheterotrophic condition (Table 3), implying that the function of these proteins is not essential in darkness. Moreover, iTRAQ observations demonstrated an accumulation of two extra FDPs (Flv1B, Flv3B) in the heterocyst-enriched cell fractions of N_2_-fixing, filamentous *Anabaena* sp. PCC 7120 and *Nostoc punctiforme* [28,29].

**Table 2:** Expression of *flv* genes in *Synechocystis* sp. PCC 6803 under various environmental conditions.

		Conditions	Log2 fold change				Database	Data accession	References
			*flv1*	*flv3*	*flv2*	*flv4*			
**Circadian**		From darkness (12h) to light (4h)			1.64		KEGG	ex0001365/70 & ex0000868/73	[30] Kucho *et al*., 2005
		From darkness (12h) to light (12h)			0.98	0.53	KEGG	ex0001377/82 & ex0000880/5	[30] Kucho *et al*., 2005
		From darkness (12h) to light (24h)			−0.89		KEGG	ex0001395/400 & ex0000898/903	[30] Kucho *et al*., 2005
		From darkness (12h) to light (28h)				−0.67	KEGG	ex0001401/6 & ex0000904/09	[30] Kucho *et al*., 2005
		From darkness (12h) to light (32h)			−0.76		KEGG	ex0001407/12 & ex0000910/5	[30] Kucho *et al*., 2005
		From darkness (12h) to light (44h)			−0.90		KEGG	ex0001425/30 & ex0000928/33	[30] Kucho *et al*., 2005
**Dark/Light**		From light to darkness (30 min)		−0.81	−2.64	−1.62	GEO	GSE45667	[31] Lehmann *et al*., 2013
		From light to darkness (5.5 h)			−2.88	−2.03	GEO	GSE45667	[31] Lehmann *et al.*, 2013
		From light to darkness (11.5h)		−0.68	−2.78	−1.85	GEO	GSE45667	[31] Lehmann *et al.*, 2013
		From light to darkness (1h)		−079			GEO	GSE16162	[22] Mitschke *et al.*, 2011
		From darkness to light (30 min)		−0.68	−3.54	−2.85	GEO	GSE45667	[31] Lehmann *et al.*, 2013
		From darkness to light (5.5h)		−0.88	−3.30	−2.56	GEO	GSE45667	[31] Lehmann *et al.*, 2013
**Light regime**		HL_15min			0.54	0.70	ArrayExpress	E-TABM-333	[32] Singh *et al.*, 2008
		HL_1h			0.93	1.00	ArrayExpress	E-TABM-333	[32] Singh *et al.*, 2008
		HL_2h			2.04	2.40	ArrayExpress	E-TABM-333	[32] Singh *et al.*, 2008
		HL_3h			0.64	0.81	ArrayExpress	E-TABM-333	[32] Singh *et al.*, 2008
		HL_4h			1.26	1.47	ArrayExpress	E-TABM-333	[32] Singh *et al.*, 2008
		HL(2)_15min			2.91	2.42	KEGG	ex0000140/3 & ex0000160/1	[33] Hihara *et al.*, 2001
		HL(2)_1h				0.55	KEGG	ex0000144/7 & ex0000152/3	[33] Hihara *et al.*, 2001
		HL			1.40	0.94	GEO	GSE16162	[22] Mitschke *et al.*, 2011
		3h illumination with red and blue light			−3.42	−2.22	ArrayExpress	E-TABM-339	[34] Singh *et al.*, 2009
		6h illumination with red and blue light			1.14	0.74	ArrayExpress	E-TABM-339	[35] Singh *et al.*, 2009
**Ox-stress**		Methyl viologen_high light	−0.93		1.28	1.36	KEGG	ex0001349,54,55	[36] Kobayashi *et al.*, 2004
		Methyl viologen_moderate light	1.12				KEGG	ex0001441/4	[36] Kobayashi *et al.*, 2004
		15 min treatment with 3mM H2O2	0.72		−2.12	0.50	GEO	GSE3703	[37] Houot *et al*., 2007
		30 min treatment with 3mM H2O2	1.28	0.73		0.70	GEO	GSE3703	[37] Houot *et al*., 2007
**Cold**		22C_20min		−0.66			KEGG	ex0000002/3_ex0000012/3_14/5	[38] Suzuki *et al.*, 2001
		24C_20min		−1.20		−0.55	KEGG	ex0001878/9	[39] Prakash *et al*., 2010
		24C_60min		−0.86		−0.66	KEGG	ex0001880/1	[39] Prakash *et al*., 2010
		24C_180min		−1.15		−0.93	KEGG	ex0001882/3	[39] Prakash *et al*., 2010
		22C_20min_(2)		1.17	−1.28		KEGG	ex0001839/40	[40] Panichkin *et al*., 2006
**Heat**		heat_30min	0.59	0.66		0.62	GEO	GSE21133	[41] Rowland *et al*., 2010
		heat_1h	0.68	0.67		0.81	GEO	GSE21133	[41] Rowland *et al*., 2010
		heat_2h	0.69	0.59		0.76	GEO	GSE21133	[41] Rowland *et al*., 2010
		heat_4h	0.57			0.76	GEO	GSE21133	[41] Rowland *et al.*, 2010
		heat_8h	0.60	0.60		0.75	GEO	GSE21133	[41] Rowland *et al.*, 2010
**C-regime**		Ci_depletion		1.39	3.07	1.80	GEO	GSE16162	[22] Mitschke *et al*., 2011
		CO2_limitation_1h				−0.55	GEO	GSE1695	[42] Wang *et al.*, 2004
		CO2_limitation_3h			0.66	−0.56	GEO	GSE1695	[42] Wang *et al*., 2004
		CO2_limitation_3.3h		1.29	7.26	5.70	GEO	GSE1695	[42] Wang *et al.*, 2004
		CO2_limitation_6h		2.00	7.16	5.62	GEO	GSE1695	[42] Wang *et al.*, 2004
		CO2_limitation_12h		2.15	6.95	5.61	GEO	GSE1695	[42] Wang *et al*., 2004
		CO2_limitation_24h		2.44	6.88	6.51			[43] Eisenhut *et al*., 2007
		high_CO2_24h_vs_low_CO2_3h		−1.00	−3.77	−3.27	GEO	GSE31672	[44] Hackenberg *et al*., 2012
		high_CO2_vs_low_CO2_24h		−2.29	−5.09	−4.32	GEO	GSE31672	[44] Hackenberg *et al.*, 2012
**Cd-Zn**		Cd_15min			1.07		GEO	GSE3682	[37] Houot *et al.*, 2007
		Cd_1.5h	−0.67				GEO	GSE3682	[37] Houot *et al*., 2007
		Cd_3h			0.81	−1.05	GEO	GSE3682	[37] Houot *et al*., 2007
		Cd_5h			0.79	1.34	GEO	GSE3682	[37] Houot *et al*., 2007
		Cd_6h			0,71	1,64	GEO	GSE3682	[37] Houot *et al.*, 2007
		Cd_16h					GEO	GSE3682	[37] Houot *et al*., 2007
		Zn_excess_240min	−0.53		2.39	1.56	GEO	GSE3716	[37] Houot *et al*., 2007
**Fe**		shift from 2mM to 0.5 μM Fe- 96h		−0.58	−1.77	−0.94	GEO	GSE3717	[37] Houot *et al.*, 2007
		shift from 1mM to 0.5 μM Fe- 96h	0.79		1.88	1.64	GEO	GSE3717	[37] Houot *et al*., 2007
		Fe depletion 3h		−1.10	−5.67	−6.02	GEO	GSE39804	[45] Hernández-Prieto *et al*., 2012
		Fe depletion 12h		−1.18	−6.14	−5.80	GEO	GSE39804	[45] Hernández-Prieto *et al*., 2012
		Fe depletion 24h		−0.80	−3.23	−3.19	GEO	GSE39804	[45] Hernández-Prieto *et al*., 2012
		Fe depletion 48h		−1.17	−5.83	−6.28	GEO	GSE39804	[45] Hernández-Prieto *et al.*, 2012
		Fe depletion 72h		−0.89	−6.04	−6.08	GEO	GSE39804	[45] Hernández-Prieto *et al.*, 2012
		Fe_high_4h			1.19	−0.62	GEO	GSE3715	[37] Houot *et al*., 2007
**Novobiocin**		Novobiocin				−1.13	KEGG	ex0001825/6	[46] Prakash *et al*., 2009
		Novobiocin + heat stress	−1.50			−1.87	KEGG	ex0001831/34	[46] Prakash *et al*., 2009
		Novobiocin + low temperature		−1.03		−0.72	KEGG	ex0001827/30	[46] Prakash *et al.*, 2009
		Novobiocim treatment + salt stress	1.12	−1.10			KEGG	ex0001835/38	[46] Prakash *et al*., 2009
		NaCl		−0.71			KEGG	ex0001687/90	[47] Shoumskaya *et al.*, 2005
		0.5 M NaCl		−0.77	−3.66	−3.37	GEO	GSE37482	[48] Dickson *et al*., 2012
		Micro-oxic		0.58	1.14	1.82	GEO	GSE24882	[49] Summerfield *et al*., 2011
		Cells encapsulated in silico gel			−2.68	−0.92	GEO	GSE37482	[48] Dickson *et al*., 2012
		Acid stress			−0.49	−0.49			[50] Ohta *et al*., 2005

**Table 3:** Expression of FDPs under various environmental conditions. (The Flv1B or Flv3B proteins are marked with *.)

Conditions	Expression				Strain	Technique	Reference
	Flv1 (A) or B*	Flv3 (A) or B*	Flv2	Flv4			
CO_2_ limitation 72 h		↑ 2.41	↑ 1.64		*Synechocystis* 6803	iTRAQ shortgun	[51] Battchikova *et al*., 2010
684 mM NaCl 5 days		↑ 5.6			*Synechocystis* 6803	2D gel proteomics	[52] Fulda *et al.*, 2006
Chemoheterotrophic growth		↓ ~3.4			*Synechocystis* 6803	2D gel proteomics	[53] Kurian *et al*., 2006
Diazotrophic: ammonium	*↑ 2.2	^*^ ↑ 1.6	^*^↑ 1.8		*Anabaena* 7120	iTRAQ shortgun	[28] Ow *et al*., 2008
Heterocystis: vegetative cells	^*^↑ 1.8	^*^↑ 3.8		0.80	*Anabaena* 7120	iTRAQ shortgun	[28] Ow *et al*., 2008
Diazotrophic: ammonium	^*^↑ 2.46				*Nostoc punctiforme*	iTRAQ shortgun	[29] Ow *et al*., 2009
Heterocyst: diazotrophic	^*^↑ 3.44	^*^↑ 2.20			*Nostoc punctiforme*	iTRAQ shortgun	[29] Ow *et al*., 2009

#### 2.2.1. The *flv4-flv2* Operon Is Regulated by NdhR and Antisense-RNA

The *flv2* and *flv4* genes in *Synechocystis* sp. PCC 6803 are organized in a three-cistronic *flv4-sll0218-flv2* operon, hereafter designated as *flv4-2* operon and coding for Flv4, the small protein Sll0218 and Flv2 proteins. The expression of the *flv4-2* operon in the *Synechocystis* sp. PCC 6803 cells is tightly controlled. The operon is positively regulated by the transcription factor NdhR and negatively regulated by as-RNA, designated As1_flv4 [54]. Consequently, overexpression of As1_flv4 has been demonstrated to lead to a decrease in *flv4-2* mRNA levels. The transient induction of the *as1_flv4* promotor during the exposure of *Synechocystis* sp. PCC 6803 cells to an environmental challenge results in the momentary, but efficient, prevention of premature expression of *flv4-2*. This regulation avoids the potential for energy being wasted in unfavorable protein synthesis upon transient environmental changes.

## 3. FDPs and Their Physiological Roles in Oxygenic Photosynthetic Organisms

All FDPs found in oxygenic photosynthetic organisms belong to Class C (except in *Picea sitchensis*). This observation suggests that the fusion of a flavin reductase-like domain at the C-terminal might have specific roles for the function and sustainability of oxygenic photosynthesis. Overexpression and characterization of the *Synechocystis* sp. PCC 6803 Flv3 protein and it is truncated form (consisting of the C-terminal flavin-reductase-like domain) in *E. coli* have demonstrated that Flv3 functions as NAD(P)H:oxygen oxidoreductase and is able to fully reduce O_2_ to water, without the production of ROS. Surprisingly, the recombinant Flv3 protein demonstrated a higher affinity to NADH than NADPH during *in vitro* studies [55].

Since most studies of FDPs from photosynthetic organisms are focused on the proteins from *Synechocystis* sp. PCC 6803 and *Anabaena* sp. PCC 7120, in this review, the function of FDPs is discussed mainly based on two functional pairs in *Synechocystis* sp. PCC 6803 (Flv2/Flv4, Flv1/Flv3) and the extra FDPs in *Anabaena* sp. PCC 7120 (Flv1B and Flv3B).

### 3.1. The Role of Flv1and Flv3

#### 3.1.1. Mehler and “Mehler-like” Reactions

The FDPs in anaerobic species play a crucial protective role upon the transient exposure of the microorganism to O_2_. This is because of the unique capacity of FDPs to reduce O_2_ to water in a direct and safe way, without the formation of ROS [55].

Photoautotrophic organisms, like cyanobacteria, algae and plants, produce O_2_ as a by-product of their photosynthetic activity and concomitantly generate various negatively charged redox carriers in electron transfer processes occurring in the same compartment. Such combinations, especially where photosensitive pigments exist in oxic conditions, are likely to produce ROS, which are potentially hazardous to biological systems, particularly to the photosynthetic apparatus. Importantly, at lower concentrations ROS can also function as a signaling molecule to stimulate cellular defense and acclimation [56]. Hence, oxygenic phototrophic organisms need a sophisticated system to protect themselves against dangerous attacks by ROS generated during illumination of the light harvesting antenna as well as the Photosystem I (PSI) and Photosystem II (PSII) reaction centers of the photosynthetic apparatus.

Chloroplasts have developed various enzymatic (catalases, superoxide dismutase, peroxidases) and non-enzymatic (glutathione, carotenoids, tocopherol) defense systems for the efficient scavenging of ROS. Photoreduction of O_2_ to H_2_O_2_ by the photosynthetic electron transport chain was described for the first time in chloroplasts by Mehler [57,58], and is therefore known as the Mehler reaction. Later on, the primary product of photoreduction of O_2_ was identified as a superoxide anion (O_2_^–^), and H_2_O_2_ was specified as a disproportionation product resulting from the function of superoxide dismutase. H_2_O_2_ is, in turn, rapidly detoxified to water by the ascorbate peroxidase pathway. In this process, the electrons derived from water splitting by PSII subsequently flow through PSI to produce water again. Thus, it has been termed the water–water cycle, or “pseudocyclic electron flow” [59,60]. The physiological relevance of the water–water cycle in chloroplasts, however, has been the subject of heavy discussion and a clear consensus has not been reached so far. One of the reasons for this is related to methodological problems. Application of membrane inlet mass spectrometry (MIMS) and the ^18^O_2_ isotope allows differentiation between O_2_ produced by PSII and that consumed by O_2_ photoreduction. However, the precise measurement of the Mehler reaction is complicated due to concomitant O_2_ uptake by chlororespiration, the photorespiratory pathway and mitochondrial respiration. It has been proposed that the water–water cycle has a dual function [60]. When the electron transfer rate exceeds the capacity of utilization of electrons by CO_2_ assimilation, the flux of “extra” electrons from and/or downstream of PSI to O_2_ [61] may provide an organism with a protective mechanism for dissipation of excess electrons. Moreover, the Mehler reaction contributes to the generation of a proton gradient across the thylakoid membrane, thus down-regulating PSII and stimulating a rapid induction of non-photochemical energy quenching (NPQ) to dissipate excess photons but, at the same time, allowing the synthesis of ATP for cellular metabolism. Importantly, the protective function of the Mehler reaction in chloroplasts is considered possible as long as the ROS scavenging system functions properly and there is a balance between ROS production and scavenging. When this balance is disturbed, massive ROS formation leads to oxidative stress.

Compared to eukaryotic phytoplankton and plants, cyanobacteria are more sensitive to H_2_O_2_ [62,63]. The reason for this could be a poor ROS scavenging system. In line with this, cyanobacteria apply a different strategy from that of the plant-type Mehler reaction (herein referred to as “true” Mehler). They can photoreduce O_2_ with electrons mediated by PSI by means of soluble Flv1 and Flv3 proteins without the production of ROS [55,64,65,66,67]. As opposed to the “true” Mehler reaction, this is a four-electron transfer reaction and ROS is not released during the process. Due to this decisive difference, we refer to the O_2_ photoreduction performed by FDPs in cyanobacteria as a “Mehler-like” reaction [67]. The difference in the mechanism of O_2_ photoreduction between cyanobacteria (Mehler-like reaction) and plant chloroplasts (“true” Mehler reaction) was further supported by comparative studies of light induced O_2_ uptake between intact *Synechocystis* sp. PCC 6803 cells and the thylakoids of pea (*Pisum sativum*), revealing significant differences in the fractionation slopes of the three stable oxygen isotopes [65].

In *Synechocystis* sp. PCC 6803 the extent of Flv1 and Flv3-mediated electron flow to O_2_ varies depending on carbon and light regime. Under ambient CO_2_ and high light (300–500 µmol photons m^–2^ s^–1^) conditions about 20% of electrons originating from water-splitting PSII can photoreduce O_2_ via Flv1 and Flv3 [66]. Light-induced electron flux to O_2_ in the high CO_2_ maintained cells has been reported to be 15% to 30% [64], and the isotopic fingerprint results obtained with three stable oxygen isotopes demonstrated that electron flow to O_2_ can be as high as 40% of gross O_2_ evolution [65]. Such differences in reported results are likely due to different experimental set-ups and physiological states of the cells. Moreover, accurate experiments with high CO_2_ maintained ∆*flv3* mutant cells revealed that about 6% of electrons originating from PSII water splitting is directed to O_2_, most likely due to activity of dark respiratory terminal oxidases in the light [65]. This hints that respiratory terminal oxidases also can contribute to O_2_ photoreduction under specific conditions. However, it is plausible that in the presence of powerful Flv1/Flv3 proteins and under standard growth conditions this contribution does not occur.

Despite the fact that Flv1/Flv3 can redirect a considerable amount of electrons to O_2_ under high-light conditions, the ∆*flv1* and ∆*flv3* single mutants and the ∆*flv1/*∆*flv3* double mutant do not demonstrate strong high light sensitivity and their net photosynthetic activities are similar to those of wild-type cells [2,64,66,67]. One possible explanation for this phenomenon might be the compensatory effect of other alternative electron transport routes.

#### 3.1.2. Flv1 and Flv3 Proteins are Crucial for the Survival of Cyanobacteria under Fluctuating Light Intensities

It is conceivable that Flv1 and Flv3 proteins play an important role during the induction of photosynthesis after a dark period, prior to the activation of Calvin–Benson cycle enzymes. This assumption is corroborated by the fact that at the onset of light exposure, after a long dark acclimation, the ∆*flv1* and ∆*flv3* mutants exhibit a lag-phase in oxidation of P700 due to a strong acceptor-side limitation of PSI and highly reduced plastoquinone (PQ)-pool. The reduced PQ-pool subsequently relaxes to normal wild-type level due to light-activation of the CO_2_ fixation machinery [64], and most likely, up-regulation of alternative electron transfer routes, which keep the PQ-pool under strict homeostatic control [68]. Consequently, the ∆*flv1* and ∆*flv3* mutant cells cultivated under diurnal dark/light regime do not show a growth phenotype. 

Nevertheless, the natural light environment of photosynthetic organisms is more challenging than diurnal dark/light cycles, consisting of highly dynamic fluctuations of light intensity and quality. Natural aquatic systems are characterized by high frequency light fluctuations due to the focusing and defocusing of sun light by surface waves. An indispensable role for the Flv1 and Flv3 proteins acting as a strong electron sink becomes evident only under fluctuating light conditions, when low background light is regularly interrupted with high light pulses [67]. Under fluctuating light the Flv1, Flv3 and Flv1/Flv3 deficient mutants show extreme and, importantly, regularly repeated acceptor side limitation, which induces strong and regular over-reduction of electron-transport chain and a blockage of photosynthesis during the high-light phases. Indeed, the Flv1 and Flv3 proteins maintain the redox balance of the electron transfer chain in cyanobacteria and provide photoprotection for PSI under fluctuating light conditions. In line with this, the growth and photosynthesis of the Δ*flv1*(*a*) and/or Δ*flv3*(*a*) mutants of both *Synechocystis* sp. PCC 6803 and *Anabaena* sp. PCC 7120 become arrested, ultimately resulting in cell death, in the most severe and long-term fluctuating light conditions. Such phenomenon is mainly caused by PSI malfunction and concomitant oxidative stress induced by ROS generated during abrupt short-term increases in light intensity, as evidenced by high carbonylation levels of proteins. However, it is also likely linked to a shortage of ATP, as evidenced by the low light-induced energization of the membrane [67].

#### 3.1.3. Cooperation of the FDP Mediated Mehler-Like Reaction and the Photorespiratory Pathway in Cyanobacteria

Besides the Mehler reaction and dark respiration, photorespiration also consumes O_2_ in oxygenic photosynthetic organisms. Photorespiration is based on the oxygenation activity of RuBisCO, the key enzyme of photosynthetic CO_2_ assimilation, which binds either CO_2_ or O_2_ in the active site, depending on the partial pressure of these gases. During evolution, many cyanobacteria have developed unique carbon concentration mechanisms (CCM) to facilitate the carboxylation of RuBisCO and to be able to grow under ambient Ci-limiting conditions [15,16,69,70]. Therefore, for many decades it was believed that photorespiration, the oxygenation of RuBisCO, does not occur in cyanobacteria [71]. More recently, however, an active photorespiratory metabolism was also discovered in the cyanobacterium *Synechocystis* sp. PCC 6803 [66,72,73,74]. 

The photoreduction of O_2_ by Flv1 and Flv3 proteins was long seen as the major obstacle to the direct monitoring of photorespiratory gas-exchange in cyanobacteria. Indeed, the capacity of the Mehler-like reaction in *Synechocystis* sp. PCC 6803 seems to be very high. In *Synechocystis* sp. PCC 6803 cells under Ci-limitation, which favors oxygenation and suppresses the carboxylation of RuBisCO, about 60% of the electrons originating from PSII can be transferred to O_2_ during the dark-light transition [66]. Iodoacetamide (IAC), a widely used inhibitor of CO_2_ fixation and photorespiration [64,75] was found to have a stimulatory effect on the photoreduction of O_2_ under Ci-limitation, perhaps by suppressing the CO_2_ assimilation and thereby stimulating the electron flux to Mehler-like reaction to a larger extent. Nevertheless, it is important to note that the occurrence of the “true”-Mehler reaction in wild-type *Synechocystis* sp. PCC 6803 cells under these conditions cannot be fully excluded.

Application of ^18^O_2_ labeling and severe Ci-limitation of cells lacking the Mehler-like reaction, Δ*flv1*/Δ*flv3*, has revealed a strong photoreduction of O_2_, whereby 40%–60% of electrons are transferred from the photosynthetic electron transfer chain to O_2_. The majority of this O_2_ photoreduction was carried out by photorespiration, since it could be inhibited by the application of IAC [66]. This observation confirms that both the FDP-mediated Mehler-like reaction and the photorespiratory metabolism are effective sinks for electrons under conditions of Ci-limitation. In line with these results, cooperation between the photorespiration and the Mehler-like reaction was revealed in a double mutant defective in both Flv3 and the glycine decarboxylase complex subunit GsvT, which is involved in one of the photorespiratory pathways. The double mutant Δ*flv3/*Δ*gcvT* could not be segregated completely and demonstrated a high-light-sensitive phenotype [76]. About 25% of O_2_ photoreduction in the Δ*flv3* and Δ*flv1* cells has been found to be insensitive to IAC, which might belong to uninhibited fraction of RuBisCO, or possibly to the true Mehler reaction.

Based on an increasing number of reports, it is becoming clear that the Mehler-like reaction has the potential to function as an efficient sink of electrons and thereby to dissipate excess electrons from the photosynthetic electron-transfer chain of *Synechocystis* sp. PCC 6803, in cooperation with photorespiration. Nevertheless, it is likely that cyanobacteria with efficient CO_2_ concentrating mechanisms and Flv1/Flv3 proteins do not frequently use photorespiratory pathways at full capacity. 

### 3.2. Flavodiiron Proteins in Filamentous Heterocystous Cyanobacteria

The Basic Local Alignment Search Tool (BLAST) and genome sequence analyses have demonstrated that filamentous heterocystous cyanobacteria contain four to six genes encoding FDPs (Table 1). *Nostoc punctiforme* is the only exception, showing five *flv* genes. In *Anabaena* sp. PCC 7120, a N_2_-fixing filamentous heterocystous model cyanobacterium, two genes, *all4444* and *all4446*, share high sequence similarity with *flv2* and *flv4* of *Synechocystis* sp. PCC 6803, respectively, and are designated in the same way (*flv2* and *flv4* of *Anabaena* sp. PCC 7120). The *flv2* and *flv4* genes form an operon, with *all4445* in between ([2], Table 1) and demonstrate a drastic increase in transcript abundance upon CO_2_ limitation [24]. These features strongly suggest that the proteins encoded by the *flv2* and *flv4* genes in *Anabaena* sp. PCC 7120 might have a role in the photoprotection of PSII, similar to the corresponding proteins in *Synechocystis* sp. PCC 6803 (see Section 3.3).

Four other *Anabaena* sp. PCC 7120 genes: *all3891, all0177*, *all3895* and *all0178* (hereafter designated as *flv1a*, *flv1b*, *flv3a* and *flv3b*, respectively) are homologous to *sll1521* and *sll0550* (*flv1* and *flv3*) of *Synechocystis* sp. PCC 6803. The genes encoding Flv1A and Flv3A proteins are organized in the *Anabaena* sp. PCC 7120 genome as *flv3a-*3ORFs-*flv1a* and only expressed in vegetative cells [24]. Transcript levels of *flv1a* and *flv3a* are regulated by CO_2_ concentration and light intensity, similar to *flv3* in *Synechocystis* sp. PCC 6803 [24]. The *Anabaena* sp. PCC 7120 mutants deficient in Flv1A and Flv3A proteins demonstrate a strong bleaching phenotype under fluctuating light conditions, which is similar to the *Synechocystis* sp. PCC 6803 Δ*flv1* and Δ*flv3* mutants [67]. Taken together, it is conceivable that *Anabaena* sp. PCC 7120 Flv1A and Flv3A proteins also function in a cyanobacterial Mehler-like reaction, reducing O_2_ to water in vegetative cells.

As mentioned above, the two “extra” genes, *flv1b* and *flv3b,* form an operon in *Anabaena* sp. PCC 7120 [19] (Table 1). These genes are transcribed under N_2_-fixing conditions, and the respective proteins are localized exclusively in heterocysts [18,24,26,53] (Table 3). These records, together with a finding of Milligan and co-workers [77], strongly support the idea that light-induced O_2_ uptake in heterocysts of N_2_-fixing cyanobacteria could play an important role in the protection of nitrogenase. Indeed, a recent paper demonstrated that the Flv3B protein is responsible for the light-induced reduction of O_2_ in heterocysts, and participates in the maintenance of a micro-oxic environment inside heterocysts for the proper function of the N_2_-fixing machinery under the light [18]. 

Importantly, the Flv1B mutant has been shown not to contribute to the light-induced O_2_ uptake in heterocysts and growth of the ∆*flv1b* mutant did not differ from that of wild-type *Anabaena* sp. PCC 7120. Thus, the function of the Flv1B protein in heterocysts remains to be elucidated. Although terminal oxidases do not contribute to light-induced O_2_ uptake in heterocysts, as demonstrated in Δ*flv3b*, they are likely responsible for the constitutive level of O_2_ consumption independently on light or dark conditions [18]. In line with this, the mutant strain lacking Flv3B demonstrated significantly increased transcript amounts of *coxA3*, which is part of the operon encoding the heterocyst-specific terminal oxidase. Genes encoding lactate oxidase, expected to reduce O_2_, as well as rubrerythrin and Mn-catalase, which both reduce H_2_O_2_ were also upregulated [18]. 

### 3.3. The Role of Flv2–Flv4

Compared to *Synechocystis* sp. PCC 6803 Flv1 and Flv3 proteins studied both *in vitro* and *in vivo* since 2002 [55,64,65,66,67,76] the Flv2 and Flv4 proteins have received little attention, and it has only been recently that they have been recognized as important players in PSII photoprotection [2,54,78,79,80]. The ∆*flv4* mutant cells lacking all proteins encoded by the whole operon (Flv2, Sll0218 and Flv4 proteins) demonstrate slow growth and a reduced level of PSII centers. This mutant was also found to be susceptible to high light intensities under ambient CO_2_ (e.g., air level CO_2_), conditions that highly enhance the expression of *flv2* and *flv4* [2,78] (Table 2,Table 3). In sharp contrast to these observations, the overexpression of the *flv4-2* operon in *Synechocystis* sp. PCC 6803 resulted in improved photochemistry of PSII and the resistance of cells to high light intensity, as compared to control strains and knock-out mutants [79]. These findings clearly suggest a role for the Flv2/Flv4 heterodimer in the photoprotection of PSII [2,79]. Heterodimer organization of the Flv2 and Flv4 proteins is discussed in the Section 3.4. 

#### 3.3.1. An Alternative Electron Transfer Route from PSII to the Flv2/Flv4 Heterodimer

The Flv2 and Flv4 proteins are not involved in the photoreduction of O_2_ in *Synechocystis* sp. PCC 6803, at least under conditions studied so far [64,66]. Further investigations of the Flv2/Flv4 related photoprotection mechanism have suggested that these proteins are neither involved in state-transitions nor in OCP-related NPQ [2,79]. Detailed comparisons of PSII properties of wild type and mutant cells, either lacking the Flv2 and Flv4 proteins or overexpressing the *flv4–flv2* operon (*flv4-2*/OE), have revealed a newly identified electron transfer route functioning in close proximity to the Q_B_ site. This route could alleviate excitation pressure by channeling excess electrons to a yet unknown electron acceptor under ambient CO_2_ conditions [2,78,79]. Indeed, the Flv2/Flv4 heterodimer stabilizes forward electron transfer and increases the charge separation rate in PSII [80]. Importantly, an increased amount of singlet oxygen (^1^O_2_) and carotenoids in the ∆*flv4* mutant compared to the wild type, sharply contrasting the significantly decreased levels in the *flv4-2*/OE overexpression strain, strongly support the idea that the Flv2/Flv4 heterodimer protects the PSII complex by decreasing ^1^O_2_ production [79].

#### 3.3.2. Phycobilisomes and *flv4-2* Mediated Photoprotection

The Flv2/Flv4 heterodimer regulates energy transfer from phycobilisomes, more specifically from terminal emitters to the PSII reaction center [78,79]. This was evidenced by 77K fluorescence emission spectra, demonstrating intensification of the F685 nm peak in the ∆*flv4* mutant during phycobilisome excitation [78]. In sharp contrast to this observation, the *flv4-2/*OE mutant demonstrated a lower peak compared to the wild type or ∆*flv4* mutant, implying an improved energy transfer to PSII [79]. Importantly, the expression of the *flv4-2* operon is dependent on the presence of phycobilisomes. The mutants lacking phycobilisomes (PAL) or containing truncated phycobilisomes (CK and ApcDF) respectively have demonstrated nearly absent, or reduced amounts of the proteins encoded by the *flv4-2* operon [79]. Moreover, the deletion of the *flv4-2* operon induces disconnection of about 20% of phycobilisomes and reduces the PSII dimer to monomer ratio, showing a direct correlation between PSII dimer destabilization and PBS detachment [80]. In contrast to this, the ∆OCP mutant, deficient in blue-light induced NPQ, demonstrated a significant up-regulation of Flv2 and Flv4 protein content, indicating a particularly important function of Flv2 and Flv4 proteins when the OCP mechanism is absent [79]. 

### 3.4. Do Cyanobacterial FDPs Function as a Homodimer or Heterodimer? 

**Figure 3:**
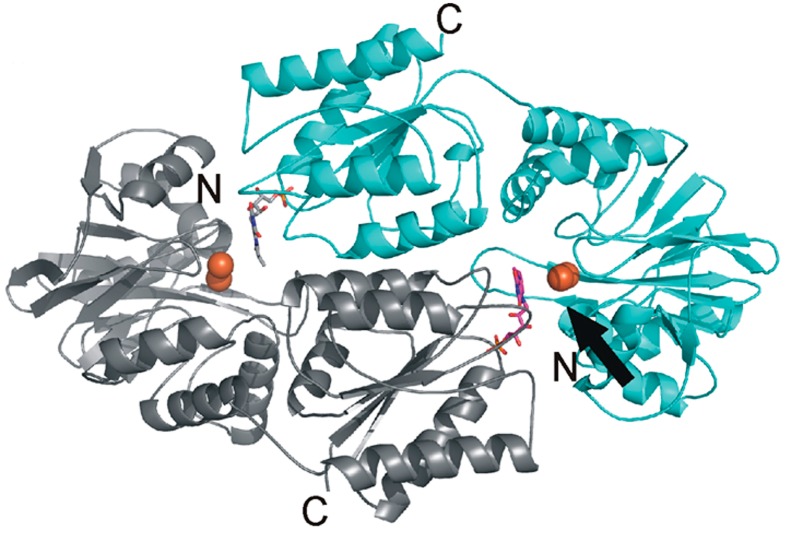
Heterodimeric organization of the Flv2 and Flv4 proteins. The functional reactive site (shown with arrow) is organized with flavin mononucleotide (FMN) (magenta) from the Flv2 monomer (gray) and diiron (orange spheres) site from the Flv4 monomer (cyan). More details in [78].

In anaerobic prokaryotes and eukaryotes, FDPs function as a homodimer or a homotetramer. In cyanobacteria, however, the organization of FDPs is more complex. The frequent co-occurrence of FDPs in cyanobacteria (as pairs or in operons) suggests their possible function as a heterodimer. Biochemical Blue Native (BN)-PAGE experiments have demonstrated that Flv2 and Flv4 proteins do indeed form a heterodimer [78]. Although Flv2 is able to form a homodimer in the absence of Flv4, complementation experiments have provided evidence that neither Flv2 nor Flv4 is physiologically functional as a homodimer. Further, constructed homology structural models (Figure 3) have demonstrated that the Flv2/Flv4 heterodimer has a more conserved active center for rapid electron transfer than that of the homodimers [78], supporting the first direct evidence of FDP heterodimer formation in *Synechocystis* sp. PCC 6803.

Unlike Flv2 and Flv4, the Flv1 and Flv3 proteins in *Synechocystis* sp. PCC 6803 have been detected in the soluble protein fraction of cells [2]. The presence of a 120 kDa protein complex in the soluble protein fraction from both the wild-type and its ∆*flv1* mutant, observed in the BN-PAGE gels probed with a Flv3-specific antibody, suggests that the Flv3 protein can organize a homodimer [66]. Moreover, the *in vitro* activity of the recombinant Flv3 protein [55] and the fact that the *flv1* and *flv3* genes are spread out in the genome, collectively support the homodimer organization of the Flv3 protein. However, the biochemical data is not corroborated by the *in vivo* functional analyses. The lack of O_2_ photoreduction in both the ∆*flv1* and the ∆ *flv3* mutants provide strong evidence for the inability of Flv1 and Flv3 to function as homodimers in the “Mehler-like” reaction in *Synechocystis* sp. PCC 6803 [64,66]. In line with the functional significance of the heterodimer, the accumulation of the Flv3 protein has been shown to be dependent on the presence of the Flv1 protein, with the amount of Flv3 significantly down-regulated in the ∆*flv1* mutant cells [66]. Nevertheless, taking into account a low transcript abundance of *flv1* and a low accumulation of the Flv1 protein in wild-type *Synechocystis* sp. PCC 6803, the possibility of Flv1 functioning as an auxiliary protein for the organization of functional Flv3 homodimer *in vivo* cannot be excluded. Indeed, further investigations are needed to clarify the details of functional dimer organization of the Flv1 and Flv3 proteins.

Likewise, the biochemical data demonstrating the organization of FDP dimers in heterocysts is still missing. However, functional data obtained thus far from the heterocysts of *Anabaena* sp. PCC 7120, strongly suggests that the Flv3B protein can function independently of Flv1B, probably as a homodimer [18].

Finally, it is worth mentioning that FDPs of oxygenic photosynthetic organisms have highly variable putative metal ligands [4,23]. The Cluster B FDPs, Flv3 and Flv4, contain canonical iron ligand residues, whereas Flv1 and Flv2 proteins from Cluster A do not contain canonical ligands at the diiron catalytic site. This raises a question about the absence of a functional metal site in the Flv1 and Flv2 proteins, in turn questioning the O_2_ reducing activity of these particular proteins [4,23].

## 4. Significance of FDPs During Evolution

FDP orthologs have been found in all genomes of obligatory anaerobic prokaryotes and some facultative microbes. Since oxygen is detrimental for strict anaerobes, mechanisms of O_2_ detoxification are crucial for their survival. It has been reported that rubredoxin:oxygen oxidoreductase, by scavenging O_2_ and preventing ROS formation *in vivo*, significantly enhances the survival of the strict anaerobe *Desulfovibrio vulgaris* under microaerophilic conditions [81]. The identification of FDPs in some microaerophilic protozoa led to the proposal of their ability to efficiently scavenge O_2_, allowing the parasites to survive in microoxic environments [6,82]. The presence of specific FDPs in oxygenic photosynthetic organisms, including almost all sequenced cyanobacteria, and some species of green algae, mosses and lycophytes, suggests that FDPs are involved in photosynthesis-related processes. Among higher plants, only *Picea sitchensis* possesses one single gene similar to *flv3* (Table 1). Analysis of the primary structure of this putative FDP showed that, unlike in other oxygenic photosynthetic organisms, the FDP of *Picea sitchensis* belongs to the Class A (Figure 1), which lacks the C-terminal flavin reductase domain typical to all other oxygenic photosynthetic organisms, thus questioning the functionality of the putative enzyme.

Photosynthetic eukaryotes and α-cyanobacteria have two (one pair of) FDPs, which are closest to *Synechocystis* sp. PCC 6803 Flv1 and Flv3, based on sequence similarity. They are probably also involved in O_2_ photoreduction (the Mehler-like reaction) to dissipate excess electrons in a harmless way. The physiological role of Flv1 and Flv3 is similar to the primary O_2_ scavenging function of FDPs in anaerobic prokaryotes. However, no FDP homologs are typical for higher plants (*Picea sitchensis* being the only exception with a single putative Class A FDP), suggesting that the cyanobacterial type Mehler-like reaction was gradually eliminated during the evolution of the green lineage, and was completely substituted by the plant-type “true” Mehler reaction, along with the development of the sophisticated ROS scavenging enzyme system. Orthologs of Flv2 and Flv4 are only found in β-cyanobacteria, indicating their limited appearance in certain subgroups and environmental niches. Originally, the Flv2 and Flv4 proteins were detected only in low Ci-acclimated wild-type *Synechocystis* sp. PCC 6803 cells [2]. However, a recent study showed a strong accumulation of *flv2* and *flv4* transcripts in a carboxysome-less mutant grown under high CO_2_ conditions, similar to the strong accumulation observed in low CO_2_ grown wild-type cells [44]. These data suggest that Flv2/Flv4 is required when electron flux to carbon fixation is largely limited.

## 5. Concluding Remarks

The crucial role of FDPs for the evolution of oxygenic photosynthesis in cyanobacteria is an intriguing discovery. The conserved *flv4-2* operon specific to β-cyanobacteria offers protection to PSII from singlet oxygen formation under strong excitation pressure by allowing electron flow from the acceptor side of PSII. The operon is expressed only under conditions where the excitation pressure on PSII becomes high, but the actual electron acceptors and the detailed electron transfer mechanism remain elusive.

Another photosystem, PSI, is also susceptible to photodamage under severe fluctuating light conditions. The Flv1 and Flv3 proteins, which are largely present in all cyanobacteria, alleviate photodamage to PSI by dissipating excess electrons down-stream of the photosynthetic electron transport chain, thus also lowering oxidative stress in the cells. The crosstalk of Flv1 and Flv3 proteins with other metabolic pathways needs more through investigation. 
